# Nature or Nurture: Is the Digestive System of the *Pontoporia blainvillei* Influenced or Determined by Its Diet?

**DOI:** 10.3390/ani14050661

**Published:** 2024-02-20

**Authors:** Carlos Tostado-Marcos, María Julieta Olocco Diz, Rosario Martín-Orti, Juan-Pablo Loureiro, Ignacio Molpeceres-Diego, Enrique Tendillo-Domínguez, Pilar Pérez-Lloret, Inmaculada Santos-Álvarez, Juncal González-Soriano

**Affiliations:** 1 Fundación Mundo Marino, Av. X 157, San Clemente del Tuyú B7105, Provincia de Buenos Aires, Argentina; tostadomarcoscarlos@gmail.com (C.T.-M.); mjoloccodiz@yahoo.com.ar (M.J.O.D.); juanploureiro@gmail.com (J.-P.L.); ignamolp11@gmail.com (I.M.-D.); enriquetd95@gmail.com (E.T.-D.); 2 Sección Departamental de Anatomía y Embriología (Veterinaria), Facultad de Veterinaria, Universidad Complutense, Avenida Puerta de Hierro s/n, 28040 Madrid, Spain; rosamart@ucm.es (R.M.-O.); pilper01@ucm.es (P.P.-L.); 3 Veterinary Histology and Pathology, Atlantic Center for Cetacean Research, University Institute of Animal Health and Food Safety (IUSA), Veterinary School, University of Las Palmas de Gran Canaria, 35413 Arucas, Spain

**Keywords:** *Pontoporia blainvillei*, anatomy, digestive system, diet, adaptations

## Abstract

**Simple Summary:**

Anatomy is considered critical to understanding the size and shape of the different systems of animals belonging to the same group or individuals that are close in the phylogenetic scale. In the case of the Franciscana dolphin or *Pontoporia blainvillei*, the digestive apparatus shows some differential characteristics compared to other dolphins or other marine mammals. In the present work, the authors try to demonstrate whether these characteristics are conditioned by the animal’s own diet or if they have more to do with certain phylogenetic adaptations. Considering that the Franciscana dolphin is an endangered species, any work that could facilitate better knowledge of these individuals has a high social, ecological, and scientific value because it will help in their care and conservation, and this is of utmost importance to implement effective management strategies for this species.

**Abstract:**

The Franciscana (also known as the La Plata River Dolphin) is a small dolphin that lives in the coastal waters of Brazil, Uruguay, and Argentina. This species is considered the most endangered marine mammal in the western South Atlantic Ocean. Anatomic dissection of the digestive system of 19 animals of different ages, including 2 neonates, 12 juveniles, and 5 adults, was performed. Parameters related to length, breadth, weight, and diameter of the digestive viscera were considered in each case. Our results show that the Franciscana dolphin presents differential characteristics in relation to several parts of the digestive system, including, specifically, the tongue, the teeth, the stomach, and the small intestine. Thus, this paper add precious information to the actual knowledge of this vulnerable marine mammal species in order to improve conservation efforts.

## 1. Introduction

The large group of marine mammals has developed numerous anatomical adaptations in response to their aquatic environment over time. Among them, cetaceans and sirenians stand out for presenting the highest degree of anatomical modifications, because they have achieved a complete adaptation to aquatic life. These two groups are distinguished, among other characteristics, by their feeding. Thus, while sirenians are the only herbivorous marine mammals, cetaceans are considered mostly carnivorous.

Cetaceans are divided into two main groups: those with teeth (Odontocetes) and those without teeth, or baleen cetaceans (Mysticetes). The Pontoporia dolphin (*Pontoporia blainvillei*), also known as the Franciscana dolphin, is classified in the suborder Odontocetes, which comprises six families and 71 species. Between them, the best known are the dolphins, but the odontocetes, or cetaceans with teeth, also include the killer whale, the sperm whale, the beaked whales, the beluga whale, the porpoises, and the narwhal. Many of them have undergone remarkable adaptations to their corresponding habitats and/or feedings, such as the loss of teeth in the upper arch (sperm whales), a small number of teeth (beaked whales), long beaks with small dorsal fins (river dolphins), or a rounded head and the absence of a beak and dorsal fin (narwhals and beluga whales). Many of these adaptations are related to their digestive system [1,2].

The Franciscana dolphin is endemic to the Atlantic coast of South America, and it represents one of the most emblematic species of the coastal waters of Argentina and Brazil [3]. Within its distribution range, up to five genetically distinct populations have been identified [4]. Like other cetaceans with coastal habits, the Franciscana is particularly vulnerable to the impact of human activities [5,6,7]. Habitat degradation, pollution, and, above all, bycatch have led to a high mortality rate of the species in recent years [8,9,10,11,12]. As a consequence, it has become the most endangered small cetacean in the southwest Atlantic Ocean [13,14], and has been classified as “Vulnerable” by the International Union for Conservation of Nature (IUCN) [15]. According to the Scientific Committee of the International Whaling Commission [16], an incidental population mortality of 1% per year is of concern in small cetacean populations, and a mortality of 2% may not be sustainable. For the Franciscana dolphin, annual mortality reaches between approximately 2% and 5% [8,11,17,18].

In Samborombón Bay (Argentina), where the species has been studied in greater detail, two distinct habitats have been observed: a brackish water estuary and an adjacent marine system. Studies have confirmed differences in the predominant prey species, but fish, especially juveniles less than 8 cm, as well as small loliginid squids constitute the preferred food in both territories [19]. In addition, evidence of predation by Franciscanas has been observed from a very early age, at approximately 2.5–3 months, when they initiate a transitional diet combining mother’s milk with solid food. Weaning in Pontoporia is gradual, with early predation on crustaceans and fish, and the lactation period is estimated to last 6–7 months [19,20].

In light of the above, the authors wonders what is meant by “diet”. In fact, what is diet? What does this term mean? What is meant when one talks about diet? The word *diet* comes from the Latin “*diaeta*”, and this in turn derives from the Greek “*dayta*”, which means regime of life. Diet, in nutrition, refers to the regular consumption of food and beverages. Therefore, a balanced diet is one that contains different types of foods in certain amounts and proportions so that requirements of calories, proteins, minerals, vitamins, and alternative nutrients are adequately met. But diet can also be defined as the total amount of food consumed by an animal or a group of animals. Moreover, diet can be considered the sum of the meals that an individual eats. This first statement includes certain parameters, such as food preferences, the prey’s size, the degree of feed hardness, and daily feeding frequency, among others.

These are the parameters that were considered in carrying out this work. Thus, the main goal of the present study was to describe in detail the digestive system of the Franciscana dolphin in trying to determine whether these anatomical characteristics could be conditioned by several factors in relation to its diet or if the digestive system of this species is modified by phylogeny.

## 2. Materials and Methods

**Figure 1:**
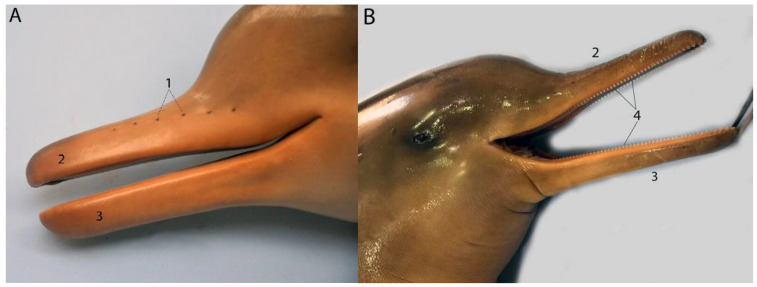
Snout of Pontoporia, external aspect: (**A**) neonate; (**B**) juvenile; (1) vibrissae; (2) maxilla; (3) mandible; (4) teeth.

This anatomical study was carried out on 19 cadavers of the species *Pontoporia blainvillei*, made up of 5 adults, 12 juveniles, and 2 neonates. The individuals were classified into three groups according to age and body development according to data published by Arruda Ramos et al. [21]. Neonates have a body length of approximately 71 cm and no teeth. The vibrissae and umbilical cord can also be observed. Juveniles include individuals with a body length up to 130 cm and the presence of developing teeth (Figure 1). Adults have a body length greater than 130 cm and the presence of developed teeth.

In all cases, except one, they were found stranded dead on the beach. The dolphin cadavers, in all cases in good conditions, were transferred to the necropsy room of the rescue and rehabilitation center Fundación Mundo Marino, where anatomical dissections of each animal were carried out. Only one neonate was found stranded alive on the beach, because quite often, sick animals go to the shore to die. It later died during the rehabilitation process. All of them belonged to the group Area FMA sub-B (1), a population that inhabits the coast of the province of Buenos Aires. In no case were the animals frozen. Dissections were carried out over a period of less than 12 h from the time the cadavers were found. When it was necessary, the cadavers was kept under refrigeration (for less than 12 h). Practically in all cases, dissections were performed according to the “Documento Técnico sobre Protocolo Nacional de Actuación para Cetáceos Varamientos de Cetáceos” published by BEVACET [22], with the animals placed in right lateral recumbency. A few cases were placed in ventral recumbency and supine recumbency in order to have a complete visualization of the digestive system’s topography. All dissections were carried out by veterinarians.

To keep the esophagus intact, the superficial musculature of the neck area was removed, exposing the trachea and esophagus. Then, a triangular incision along the inner sides of the jaws was made. The hyoid apparatus was cut, and the tongue was extracted through the intermandibular space. Then, the cardiorespiratory system was progressively separated.

To make the esophagus independent, its connections with the respiratory system (esophagus–trachea) were removed, and it was sectioned at the beginning, exactly at the level of the pharynx. To maintain the esophagus–stomach union, an eyelet-shaped cut was made around the esophageal hiatus. Finally, the entire digestive system was externalized by sectioning the most caudal part of the rectum, including the liver and the pancreas.

As mentioned before, the present study included different specimens—5 adults, 12 juveniles, and 2 neonates—each with obviously different degrees of development. Because of this, the corresponding arithmetic means of the data obtained were calculated separately to avoid deviations and erroneous data.

## 3. Results

In the Franciscana dolphin, cranial asymmetry (determined by the distance between the eye and the blowhole) was very slight, with a mean in juveniles of 9.2 cm on the right side and 7.8 cm on the left side; in neonates, it was 8 cm on the right side and 7 cm on the left side.

**Figure 2:**
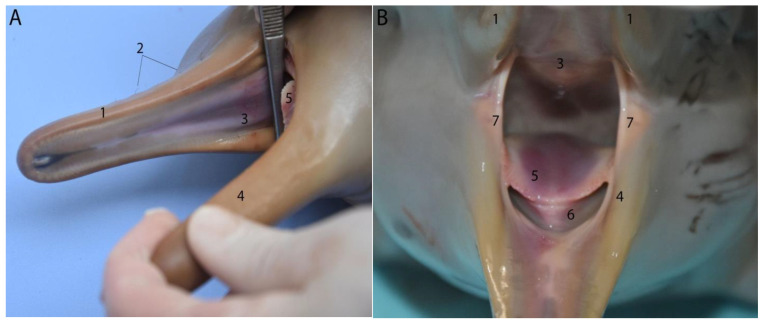
Roof and floor of the oral cavity of a neonate individual. Note the absence of teeth: (**A**) lateral view; (**B**) rostral view; (1) maxilla; (2) vibrissae; (3) hard palate; (4) mandible; (5) tongue; (6) musculature of the tongue; (7) corners of the mouth.

The snout of the Franciscana dolphin grows and lengthens as the animal ages until it reaches adulthood, as occurs in other river dolphin species (Figure 1). In neonate individuals, small vibrissae can be observed in the area of the snout (maxilla) that will disappear after a few days of life (Figure 2).

### 3.1. Oral Cavity

**Figure 3:**
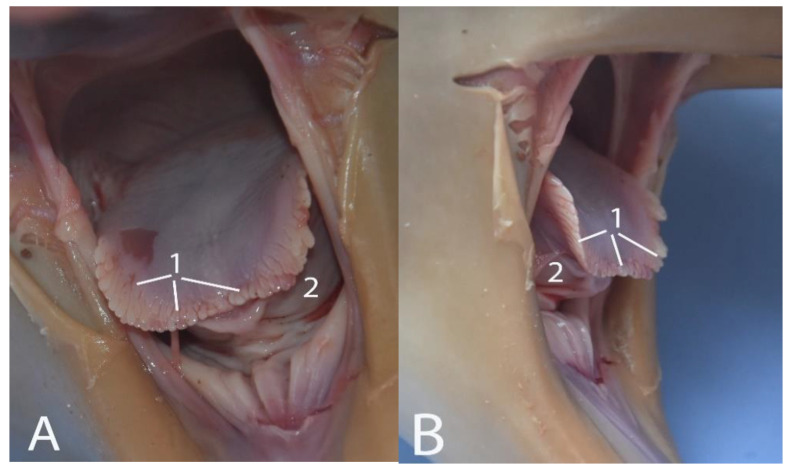
Tongue of lactating individuals: (**A**) rostral view; (**B**) lateral view; (1) anterolateral mechanical papillae; (2) tongue musculature.

The buccal cavity is elongated, and it has a large buccal opening angle (Figure 3). This is due, in part, to the telescoping of the skull (a process of rostral elongation of the dolphin skull) and also the presence of folds at the level of the corners of the mouth (folds that join the palate and the jaw) (Figure 3). The distance between the two folds is related to the size of the isthmus of the fauces. This is important, as it will determine the size of the prey that the Franciscana dolphin is able to ingest. In neonates, the maw isthmus width was 2.7 cm, and in juveniles it was 3.2 cm.

#### 3.1.1. Teeth

This species has a homodont dentition (Figure 1). The total number of teeth is high, with small variations among individuals. The total number ranges from 220 to 240. Normally, the maxilla has more teeth than the mandible (Table 1). Its morphology is conical, thin, and small in size. It has a saw-like appearance. They are very sharp teeth. Their feeding function is to press their prey, developing “ram feeding.” There is a slight variation in the length of the teeth according to the area of the snout. There is growth as they advance from the most rostral part to the caudal, reaching a maximum size in the middle of the snout approximately, and then they begin to decrease, presenting the smallest size in the most caudal area of the snout, forming a plateau structure (Table 1).

These animals are born without teeth. They do not have deciduous teeth, and they directly develop permanent teeth. They are monophyodont animals (teeth erupt only once). Eruption begins after approximately 2 months of lactation.

#### 3.1.2. Tongue

The free portion of the tongue is small, short, thick, and somewhat mobile. It occupies most of the buccal cavity proper, and its limit is marked by the corner of the mouth (Figure 2 and Figure 3). The average length is about 6 cm, with very little variation among the different age groups. There is also a slight difference in width between the base and the tip (Table 2). Neonate and juvenile individuals have anterolateral mechanical papillae arranged on both sides of the edge of the tongue apex in a single row. A clear decrease in the number of papillae was observed with age. As the individual develops, there is progressive atrophy/involution of the papillae until they disappear completely in adulthood because their functionality is attributed to the sucking process during lactation. These papillae are small, thin, and elongated. In lactating individuals, the average number of papillae was 50, with 32 in juveniles and 3 in adults.

### 3.2. Esophagus

**Figure 4:**
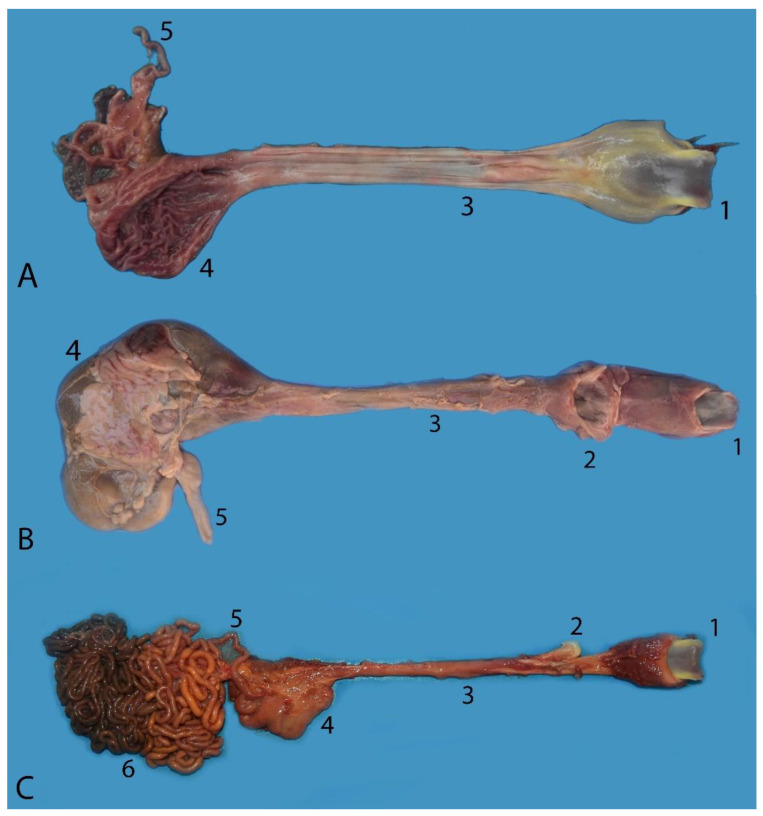
Digestive tract of a neonate individual: (**A**) opened; (**B**,**C**) closed. (1) Tongue; (2) goose beak (formed by epiglottic, arytenoid, and cricoid cartilages); (3) esophagus; (4) stomach; (5) duodenum; (6) jejunum.

**Figure 5:**
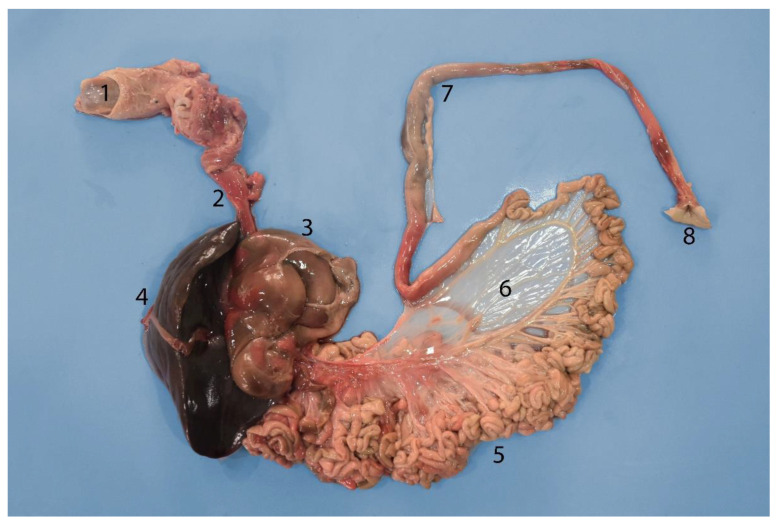
Complete digestive tract of a juvenile individual outside the abdominal cavity. (1) Tongue, (2) esophagus, (3) stomach, (4) liver, (5) small intestine, (6) mesojejunum, (7) colon, (8) anus.

The esophagus of the Franciscana is of no special anatomical interest, as its structure is similar to that of other mammals. It is a tubular organ that connects the pharynx to the stomach. It runs dorsal to the trachea and moves slightly to the left at the end of its course to cross the diaphragm at the esophageal hiatus, and it joins the stomach in the intrathoracic portion of the abdomen. The esophagus joins directly with the main stomach (Figure 4 and Figure 5). The mean length was 17 cm in neonates, 25 cm in juveniles, and 31 cm in adults. It can be observed that there is a development of the organ according to body development. As for the diameter, it is very similar throughout its length. A slight variation was observed between sections. In adult specimens, the average was 5 cm in the cranial part, decreasing to 3.5 in the middle part and widening again in the caudal part near the entrance to the main stomach, with an average of 4 cm.

### 3.3. Diaphragm

**Figure 6:**
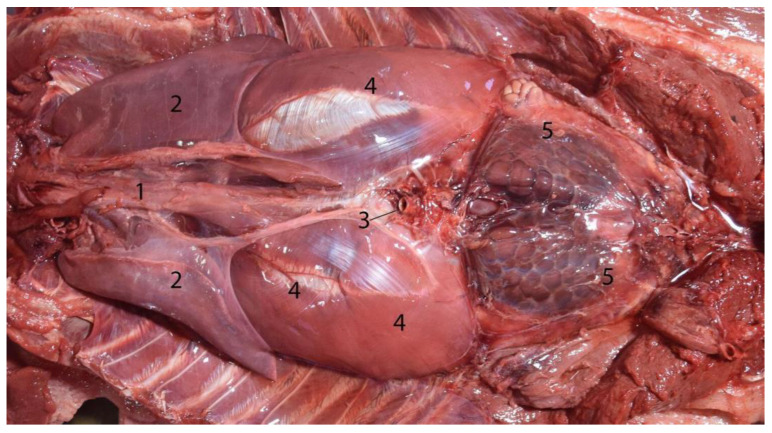
Juvenile animal positioned in prone position; dissection from dorsal to observe the topography with a dorso-ventral projection; cranial to the left, caudal to the right of the image: (1) esophagus; (2) lungs; (3) abdominal aorta; (4) diaphragm; (5) kidneys.

The diaphragm constitutes the cranial wall of the abdominal cavity and is formed by a muscular part and a tendinous or aponeurotic part. Its structure is similar to that observed in terrestrial mammals, but with some differences in this species and generally in cetaceans. It is highly developed, with a transverse/oblique orientation along a large part of the abdominal cavity. Its cranial insertion is ventral to the sternum, while the caudal part inserts near the cranial pole of the kidneys to the body of the thoracic vertebrae through the pillars of the diaphragm (Figure 6). This oblique arrangement of the diaphragm means that in these animals the intrathoracic portion of the abdomen is greater in length/depth.

### 3.4. Stomach

**Figure 7:**
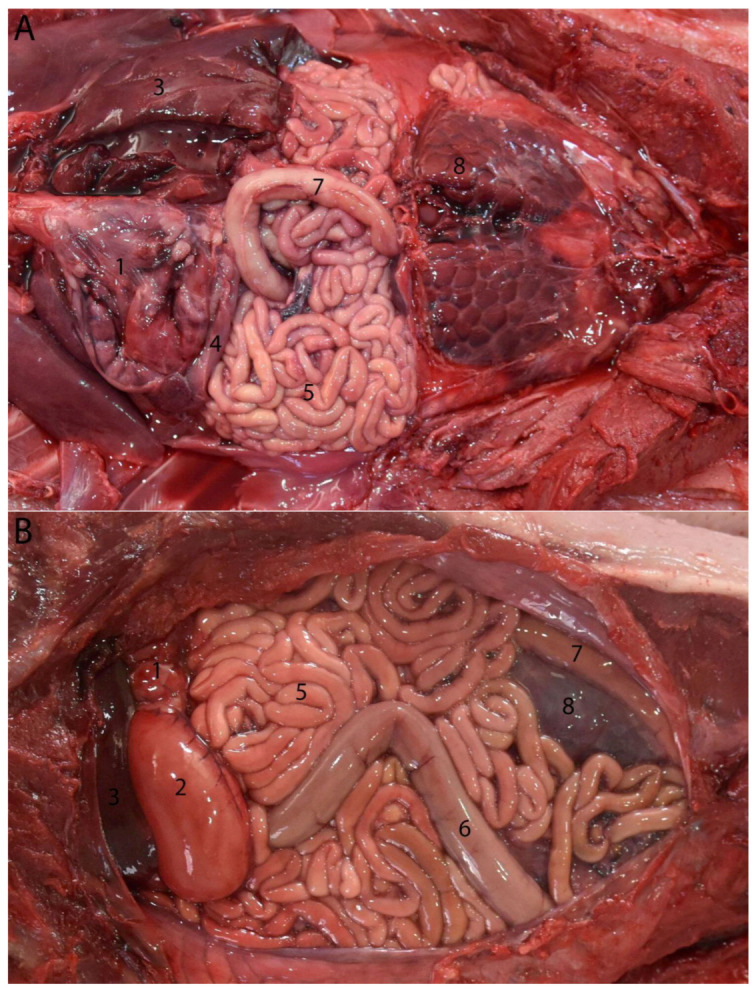
Abdominal cavity of juvenile individuals: (**A**) prone decubitus; (**B**) supine decubitus; (1) main stomach; (2) pyloric stomach; (3) liver (right lobe); (4) pancreas; (5) jejunum; (6) ascending colon; (7) descending colon (in image (**B**), it appears related to the lateral side of the left kidney, but the normal location is between both kidneys on the dorsal side of the abdomen. It was possibly displaced due to the manipulation of this individual); (8) kidney.

Topographically, the stomach is located in the cranial part of the abdominal cavity, caudal to the liver and from ventral to dorsal. It is displaced to the left side with respect to the midline. More displaced to the right side is the liver, with which it maintains a close relationship. It is formed by two chambers: chamber 1, or the main stomach, and chamber 2, or the pyloric stomach. They also have a small U-shaped structure that serves as a junction between the two chambers, called the communication channel. The main stomach is located dorsally (Figure 7A), the communication channel is located ventral and to the left of the cavity, and the second chamber, or the pyloric stomach, is located ventral and slightly to the right to give way to the duodenum (Figure 7B). All of the compartments of the stomach are of the glandular type. The dimensions of the main and pyloric stomachs are given in Table 3.

**Figure 8:**
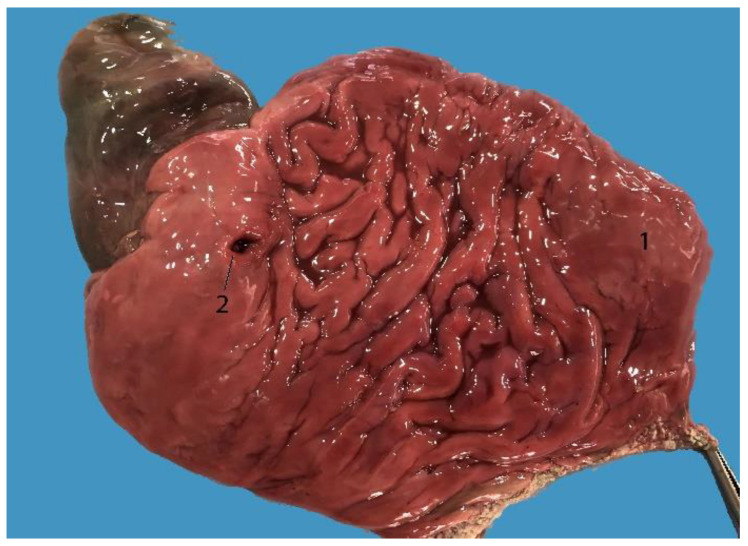
Image of the main stomach: (1) main stomach (mucosa); (2) orifice that communicates the main stomach with the communicating channel.

**Figure 9:**
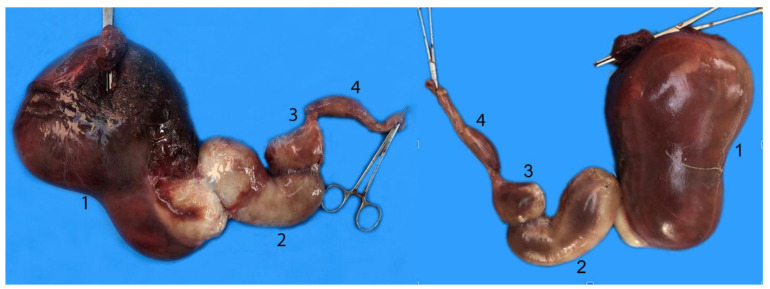
Images of stomach filled with water to obtain a better definition of the different chambers: (1) main stomach; (2) pyloric stomach; (3) duodenal ampulla; (4) duodenum.

**Figure 10:**
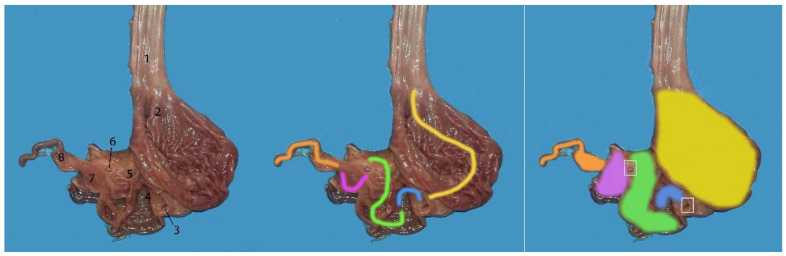
Stomach of a neonate individual. All of the chambers are opened: (1) esophagus; (2) main stomach (yellow); (3) orifice 1 (communication main stomach–communication channel) (white); (4) communication channel (blue); (5) pyloric stomach (green); (6) orifice 2 (communication pyloric stomach–duodenal ampulla) (white); (7) duodenal ampulla (violet); (8) duodenum (orange).

**Figure 11:**
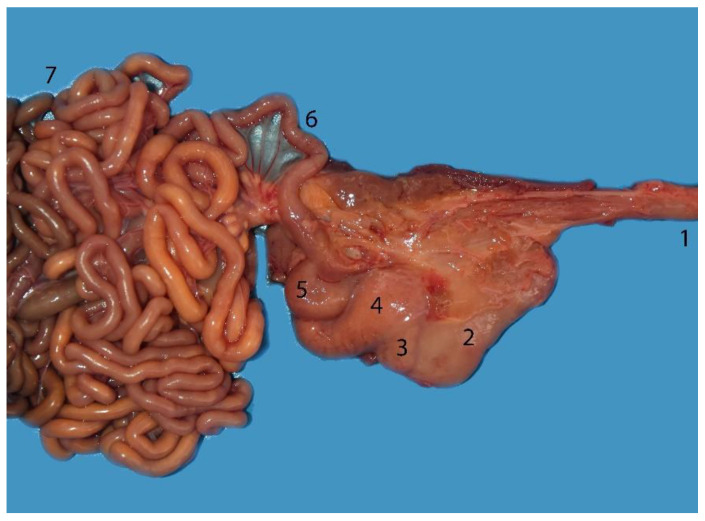
Stomach of a neonate individual: (1) esophagus; (2) main stomach; (3) communication channel; (4) pyloric stomach; (5) duodenal ampulla; (6) duodenum; (7) jejunum.

The differential characteristics shown by the Franciscana make it interesting to give a more detailed description of the stomach chambers (Figure 8, Figure 9, Figure 10 and Figure 11):

- The main stomach or chamber 1 communicates directly with the esophagus. There is no pre-stomach in this species. The mucosa of the wall of the main stomach is of a glandular type, and ridges of the mucosa can be observed. The orifice of communication with the small U-shaped structure or communication channel is narrow, about 1 cm in diameter, and more than an orifice, it has the structure of a small channel. It is located in the fundus of the stomach (Figure 8).
- Communication channel: It is a small-sized, elongated, and thin compartment that serves as a communication between the main stomach and the pyloric stomach. The interior has a U-shaped structure, starting from ventral and, after bending, advancing dorsally (Figure 9);
- The pyloric stomach or chamber 2 is smaller than the main stomach. It connects with another smaller dilatation, which corresponds to the duodenal ampulla, through an orifice whose diameter varies between 0.3 and 0.5 cm. This communication does not appear to be a sphincter but rather an orifice that allows the passage of stomach contents in a continuous manner. In contrast, the pyloric sphincter (which marks the separation between the stomach and the small intestine) is located between the duodenal ampulla and duodenum (Figure 9 and Figure 10).

### 3.5. Small Intestine

The small intestine of the Franciscana is large in size, occupying a large part of the abdominal cavity. It consists of three sections: the duodenum, jejunum, and ileum. It is complex to macroscopically determine the change between sections due to the morphological similarity between them. The diameter is quite regular throughout (Table 4), as well as the thickness of the mucosa. The criterion used to differentiate between the duodenum and the jejunum is the location of the first jejunal loop. Likewise, the change from the jejunum to the ileum is determined by the last jejunal loop. The ileum presents a straight course, without curvatures or loops. The ratio between the length of the small intestine and the body length is spectacularly high, being approximately 27:1 in juveniles.

#### 3.5.1. Duodenum and Pancreas

**Figure 12:**
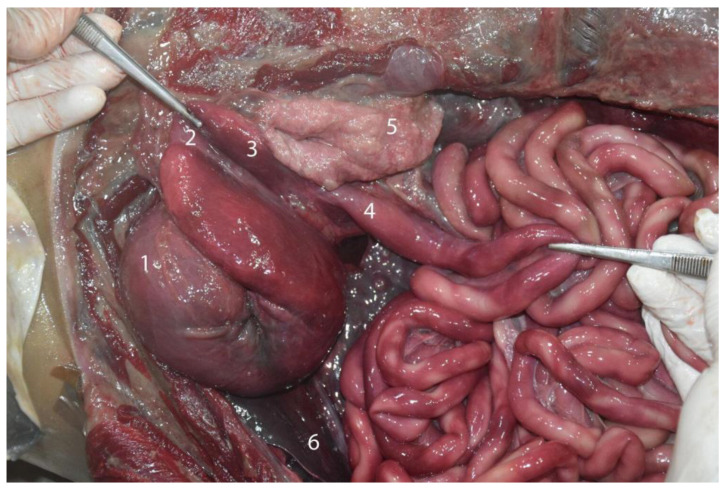
Cranial abdominal cavity of an adult individual; cranial (left)–caudal (right). By removing the stomach and pancreas, we can visualize the duodenum, located on the right side of the dorsal part of the cranial abdomen: (1) main stomach; (2) communication channel; (3) pyloric stomach; (4) duodenum; (5) pancreas (right lobe); (6) liver.

**Figure 13:**
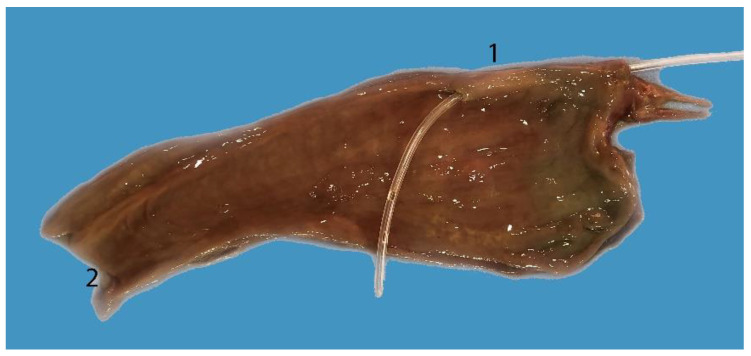
Initial portion of the duodenum opened. (1) Hepatopancreatic duct, (2) duodenum.

**Figure 14:**
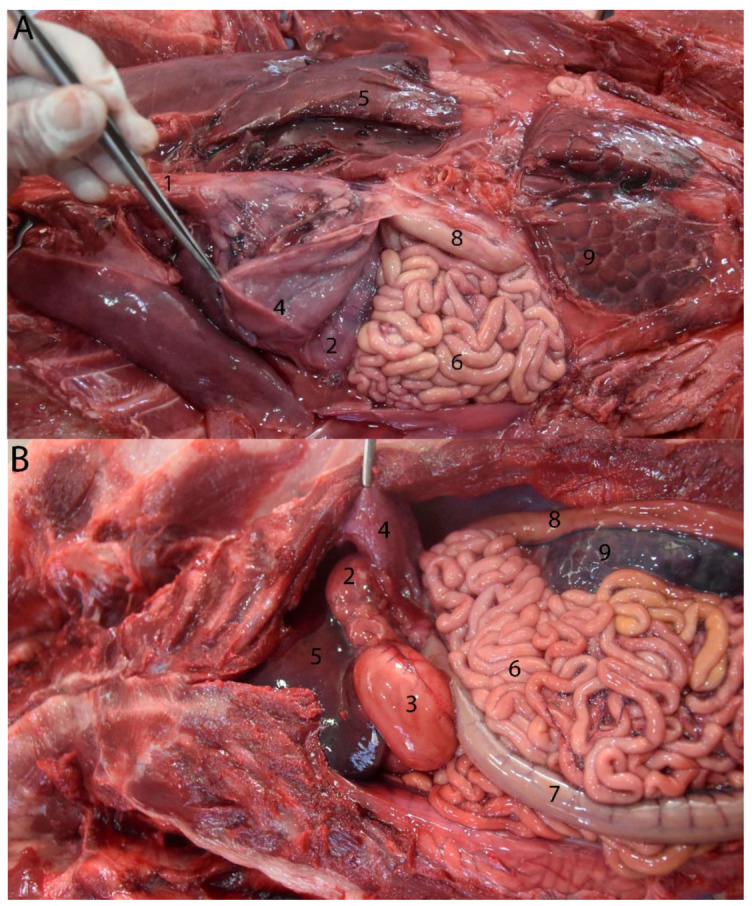

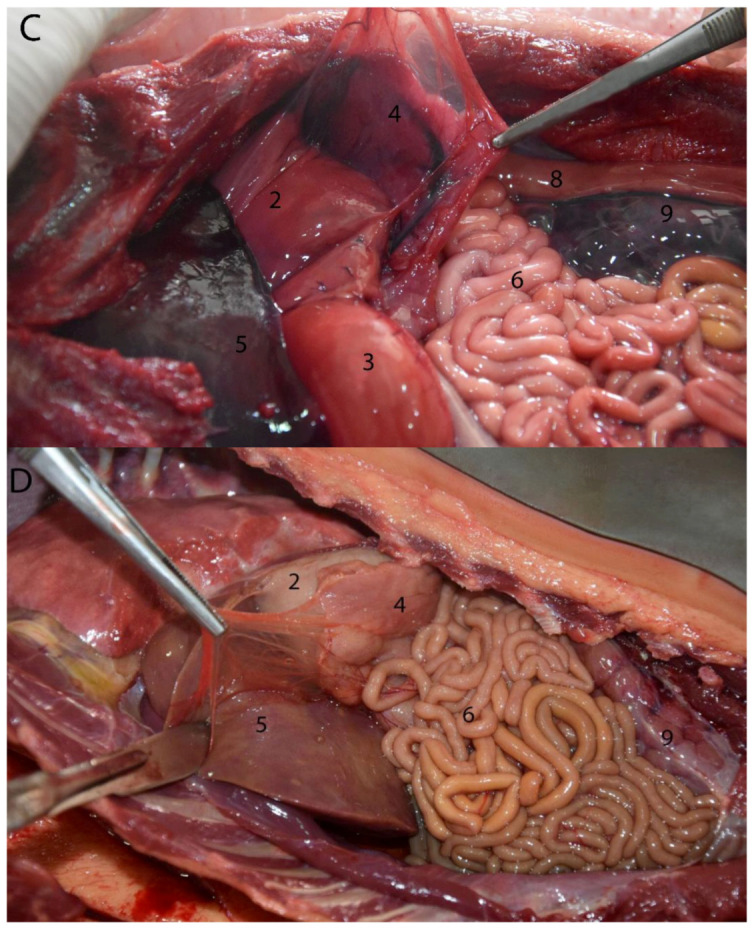
Juvenile individual: (**A**) prone decubitus; (**B**,**C**) supine decubitus; (**D**) neonate individual in supine decubitus. (1) Esophagus; (2) main stomach; (3) pyloric stomach; (4) pancreas; (5) liver; (6) jejunum; (7) ascending colon; (8) descending colon; (9) left kidney.

As mentioned above, the beginning of the duodenum presents a dilatation called the duodenal ampulla. Afterwards, it narrows until it reaches a size and lumen similar to the jejunum (Table 4). The communication between the pyloric stomach and the duodenal ampulla is through an orifice. That is, the communication between the stomach and the small intestine is not delimited by the pyloric sphincter. However, this sphincter appears between the duodenal ampulla and the next section of the duodenum (Figure 9, Figure 10, Figure 11, Figure 12, Figure 13 and Figure 14). The pancreas presents two lobes, and it is intimately related to the duodenum and stomach, as is the case in other species. The study of the pancreas was not possible in all individuals, given its high enzymatic activity and rapid post-mortem autolysis.

In the duodenum ends a duct that is common to the liver and pancreas and is called the hepatopancreatic duct. This duct runs along the wall of the duodenum from its beginning to its end inside the duodenum (Figure 13).

#### 3.5.2. Jejunum

**Figure 15:**
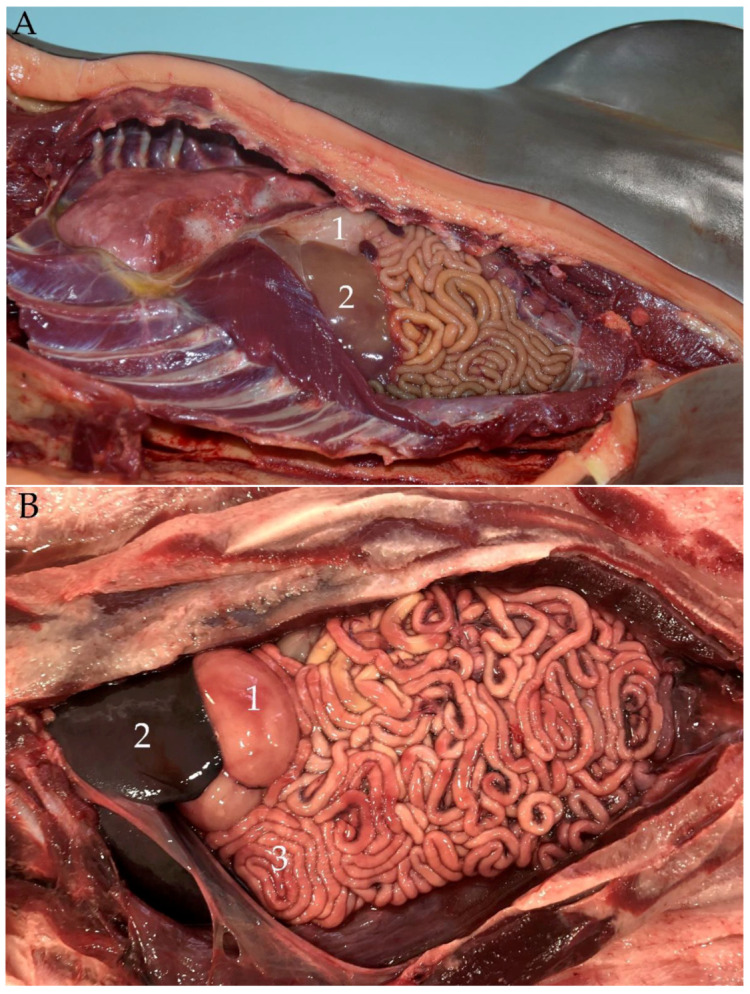

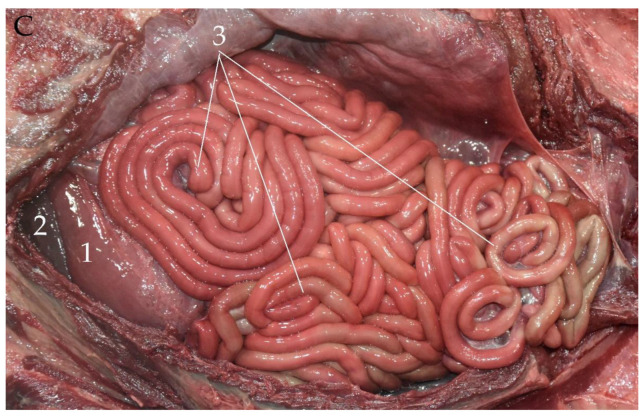
(**A**) Neonate individual in right lateral decubitus; cranial (left)–caudal (right) (without jejunal discs): (1) main stomach; (2) liver; (3) jejunum. (**B**) Juvenile individual in supine decubitus; ventral view of abdomen; cranial (left)–caudal (right): (1) main stomach; (2) liver; (3) jejunal discs. (**C**) Adult individual in right lateral decubitus–supine; cranial (left)–caudal (right): (1) stomach; (2) liver; (3) jejunal discs.

The jejunum is located caudal to the liver and stomach, occupying the entire dorsal and ventral region of the abdominal cavity. It is the longest section of the small intestine. Its length increases with the age of the animal until it reaches the adult stage (Table 4). In addition, it is worth noting a peculiar characteristic of the jejunum of this species that has not been previously described in the literature. It presents an arrangement within the abdominal cavity in the form of “discs,” with different levels of organization depending on the degree of development of the individual. A clear difference between age groups has been observed. Thus, neonate individuals presented a low level of organization, with scarce formation of these discs. As the animal develops, the same occurs with the discs. In juvenile individuals, a certain degree of organization was observed, with the appearance of some jejunal discs. Meanwhile, adult individuals presented a high level of organization of jejunal discs. A higher organization of the jejunal discs can be observed in the cranial part of the jejunum than in the caudal part (Figure 15).

#### 3.5.3. Ileum

**Figure 16:**
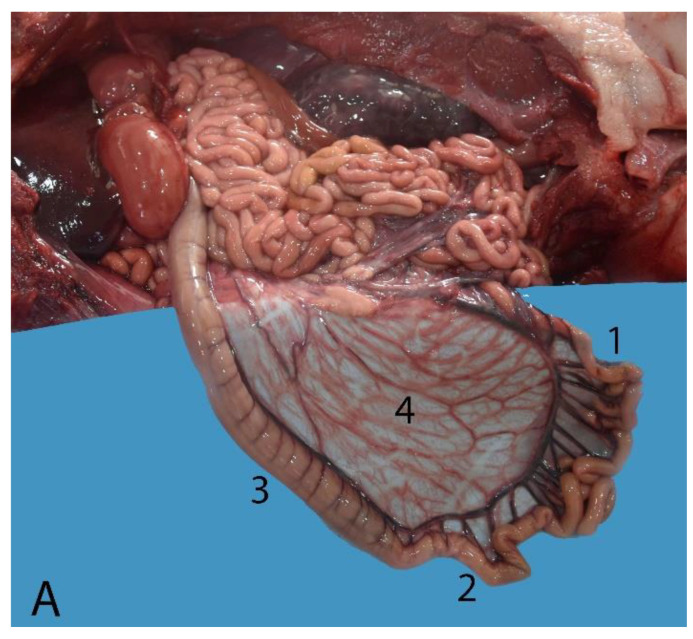

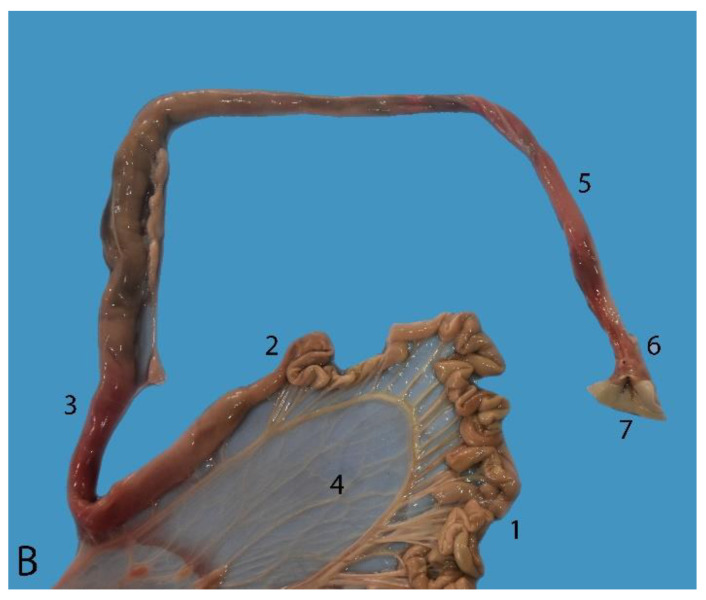
Intestine of a juvenile individual: (**A**) transition between small and large intestine; (**B**) view of the extended large intestine; (1) jejunum; (2) ileum; (3) ascending colon; (4) mesojejunum; (5) descending colon; (6) rectum; (7) anus.

**Figure 17:**
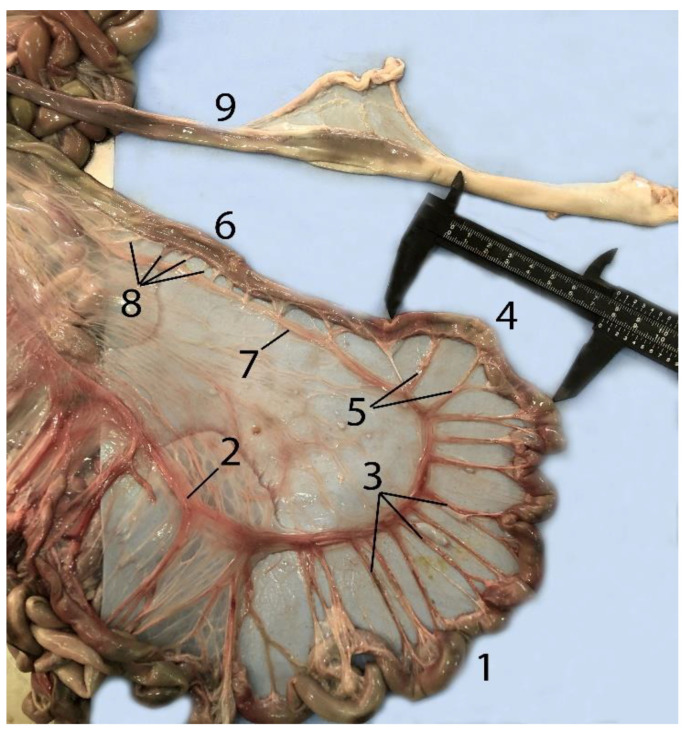
Intestine of an adult individual. The scale marks the ileum: (1) jejunum; (2) cranial mesenteric artery; (3) jejunal arteries; (4) ileum; (5) ileal arteries; (6) ascending colon; (7) caudal mesenteric artery; (8) colic arteries; (9) descending colon.

The ileum is the final section of the small intestine; it is short and of a similar diameter to the jejunum (1, 0.9, and 0.4 cm in adult, juvenile, and neonate individuals, respectively) (Table 4) (Figure 16). Its beginning is determined by the last jejunal loop. Its end is marked by the beginning of the colon (with its mucosal ridges), as well as the last ileal blood vessel (Figure 16 and Figure 17). The mean length was 9.5, 5.9, and 4 cm in adult, juvenile and neonate individuals, respectively.

### 3.6. Large Intestine

The Franciscana dolphin does not have a cecum, which makes it difficult to differentiate the change to the large intestine. No histochemical techniques were performed to demonstrate histological differences between the two sections. Thus, to differentiate the end of the ileum and the beginning of the colon, we based ourselves on the following criteria:

- Diameter: macroscopically, a slight change in size can be observed at the transition from the small to the large intestine (Table 4 and Table 5). They clearly mark the beginning of the colon and make it thicker.
- Wall thickness: on palpation, a change in the thickness of the wall between the small and the large intestine can be noted: ileum (thinner) and colon (thicker).

#### 3.6.1. Colon

**Figure 18:**
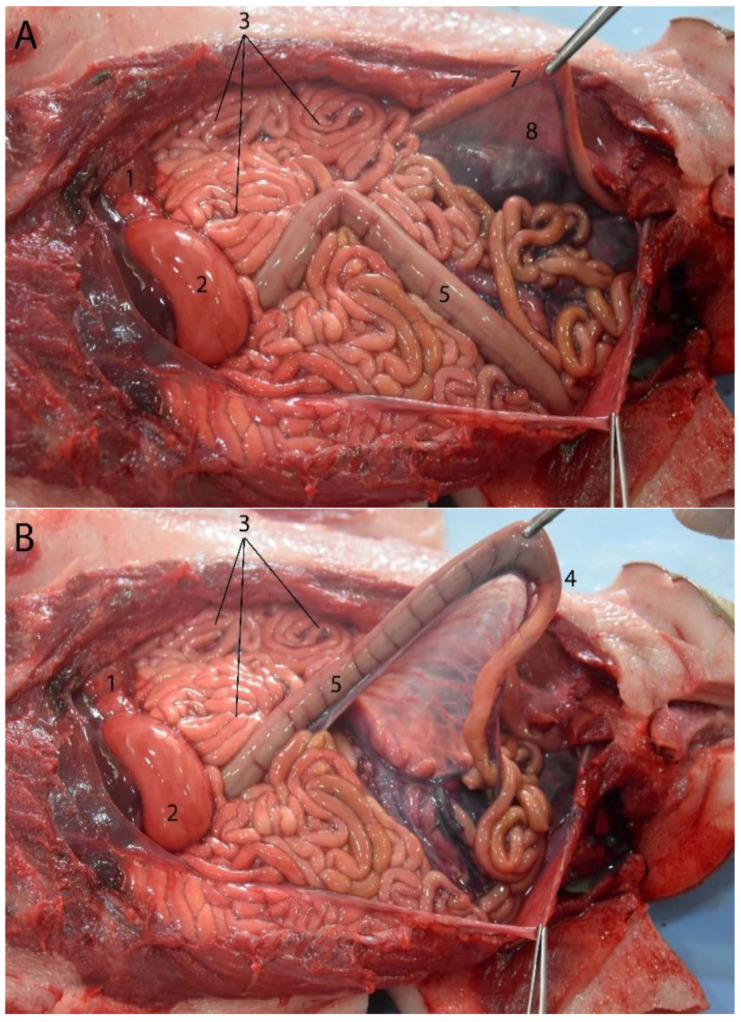

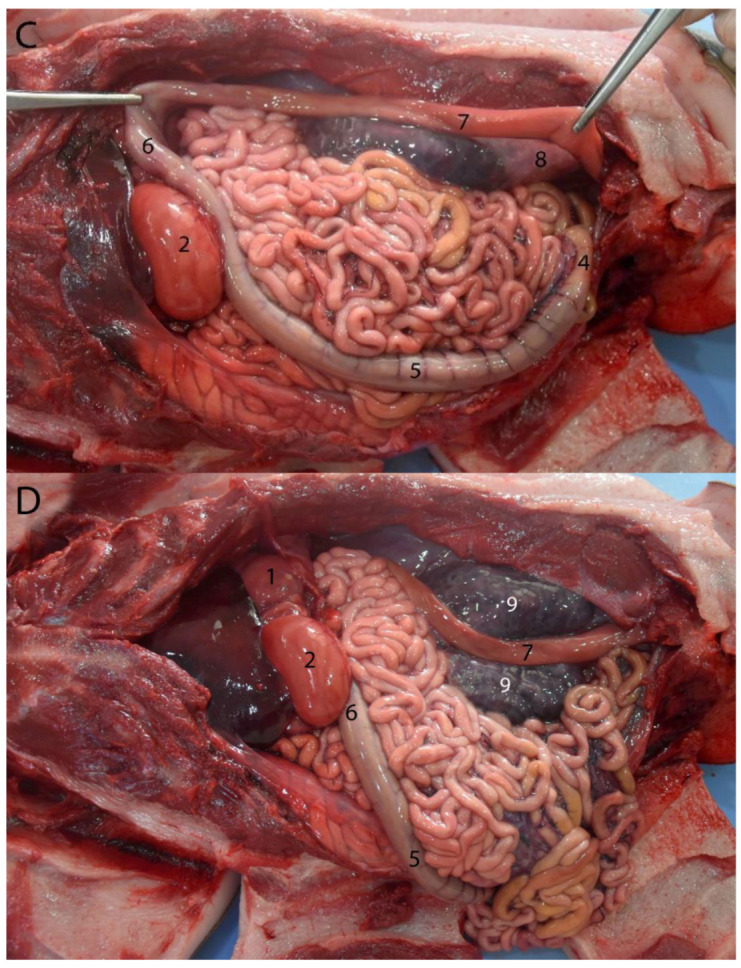
Abdominal cavity of a juvenile individual. Different planes in supine decubitus: (**A**) ventral view of the abdominal cavity; (**B**) the ascending colon is lifted to see the transition between small and large intestine; (**C**) the colon has been displaced in order to see all their parts; (**D**) the jejunum has been retired to see the course of the descending colon, between both kidneys; (1) main stomach; (2) pyloric stomach; (3) jejunal discs; (4) transition from small to large intestine (ileum–colon); (5) ascending colon; (6) transverse colon; (7) descending colon; (8) mesocolon; (9) kidneys.

The colon is divided into three sections: the ascending, transverse, and descending colon (Figure 18). The ascending colon begins in the caudal part of the abdominal cavity, and it has an ascending course located in the ventral part of the right side. Once it has reached the cranial area, where it is related to the stomach, liver, and pancreas, there is a change of direction and it runs from ventral to dorsal in an obliquely crosswise plane. This section is short, corresponding to the transverse colon. From there, the descending colon runs along the dorsal midline caudal to the abdominal cavity. It passes between the two kidneys (Figure 18D) and descends again progressively towards the ventral pelvic area until it empties into the rectum and then into the anus.

**Figure 19:**
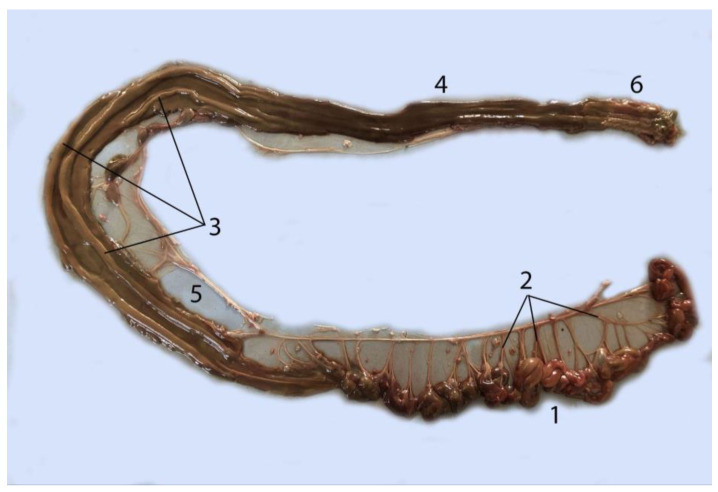
Large intestine, opened to expose the mucosa: (1) jejunum; (2) jejunal arteries; (3) folds of the colon mucosa; (4) smooth mucosa of the final stretch of the colon; (5) mesocolon; (6) rectum.

**Figure 20:**
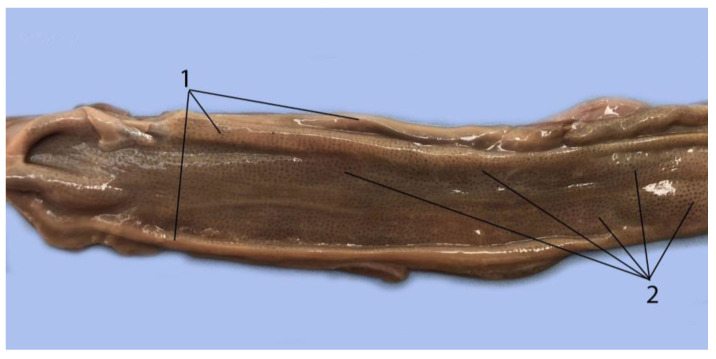
Initial section of the colon: (1) folds of the colon mucosa; (2) associated lymphatic tissue/Peyer’s patches.

In its first half, the colon presents a mucosa lined with Peyer’s plaques/lymphatic tissue associated with the intestine. In addition, in its interior, two thick folds or ridges of the mucosa stand out (Figure 19 and Figure 20). The last section of the colon, corresponding to the descending colon, has a smooth mucosa without folds. These characteristics, together with the coloration and thickness, are unique to the colon compared to the other parts of the intestine. No tapeworms coli or haustras were observed. The morphometric data of the colon are shown in Table 5.

#### 3.6.2. Rectum

**Figure 21:**
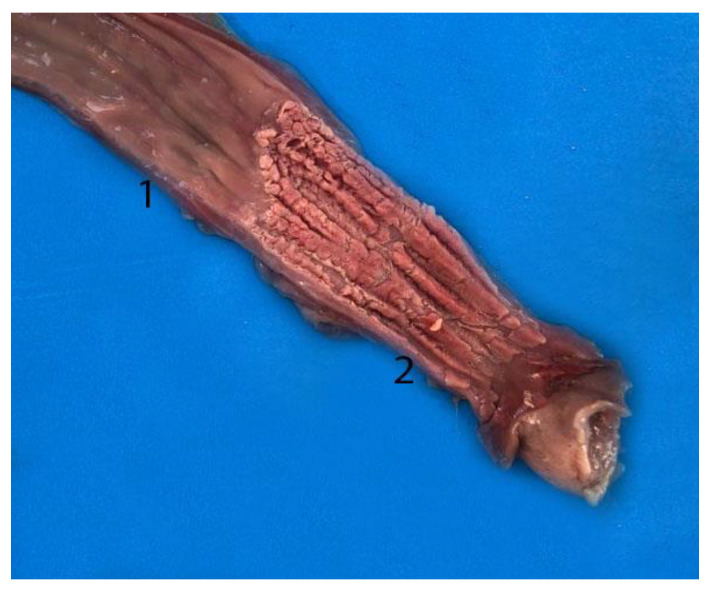
Final portion of the large intestine (opened) and anus; note the macroscopic change of the mucosa: (1) colon; (2) rectum.

In certain cases, a macroscopic change in the mucosal lining epithelium was observed in the most distal part of the large intestine. It could be compatible with the change from colon to rectum (Figure 21). In other individuals with a more advanced degree of decomposition, this difference could not be observed, possibly due to the degradation and detachment of this rougher-looking mucosa. No histochemical techniques were performed to demonstrate histological differences between the two sections.

### 3.7. Liver

**Figure 22:**
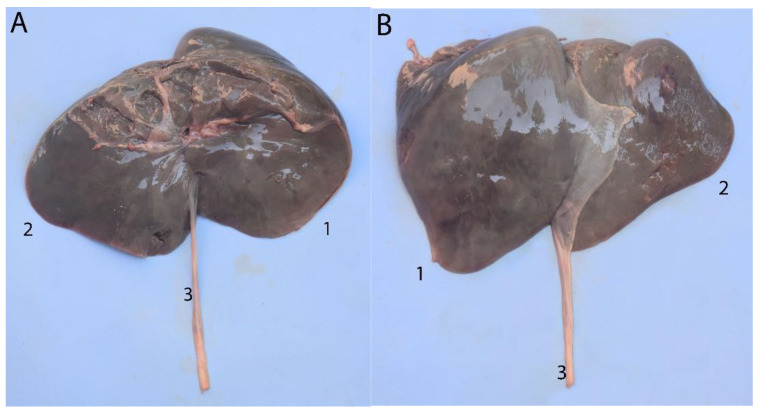
Liver: (**A**) visceral surface; (**B**) diaphragmatic surface; (1) right lobe; (2) left lobe; (3) round ligament of the liver.

**Figure 23:**
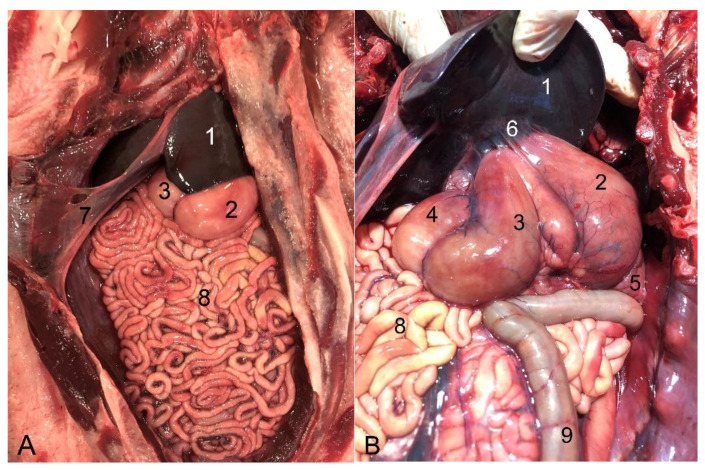
Abdominal cavity of a juvenile individual, supine decubitus: (**A**) ventral view of the abdominal cavity; (**B**) the stomach is being exposed to show the different chambers, and the jejunum has been displaced to visualize the course of the descending colon; (1) left lobe of the liver; (2) main stomach; (3) pyloric stomach; (4) duodenal ampulla; (5) pancreas; (6) lesser omentum; (7) round ligament; (8) jejunum; (9) ascending colon.

The liver is formed by two lobes divided by a shallow but well-defined fissure (Figure 22). It is located in the intrathoracic portion of the abdominal cavity. The left lobe is smaller than the right lobe (Figure 22) (Table 6) because the left side of the cavity is mostly occupied by the stomach. This lobe is located ventrally, while the right lobe is located on the right side from ventral to dorsal. On its diaphragmatic side, it presents folds that join the diaphragm, corresponding to the coronary and falciform ligaments of other mammals (Figure 22 and Figure 23). On the visceral side, it presents the hepatogastric ligament that joins it to the stomach (Figure 23). The ratio of liver to body mass ranged from 1 to 2.9% (Table 6). There was one exception, which was the infant individual that was found stranded alive and transferred to the rescue center for rehabilitation. The result was 7.5%; associated with this data, generalized jaundice was observed in the animal’s mucous membranes.

### 3.8. Spleen

**Figure 24:**
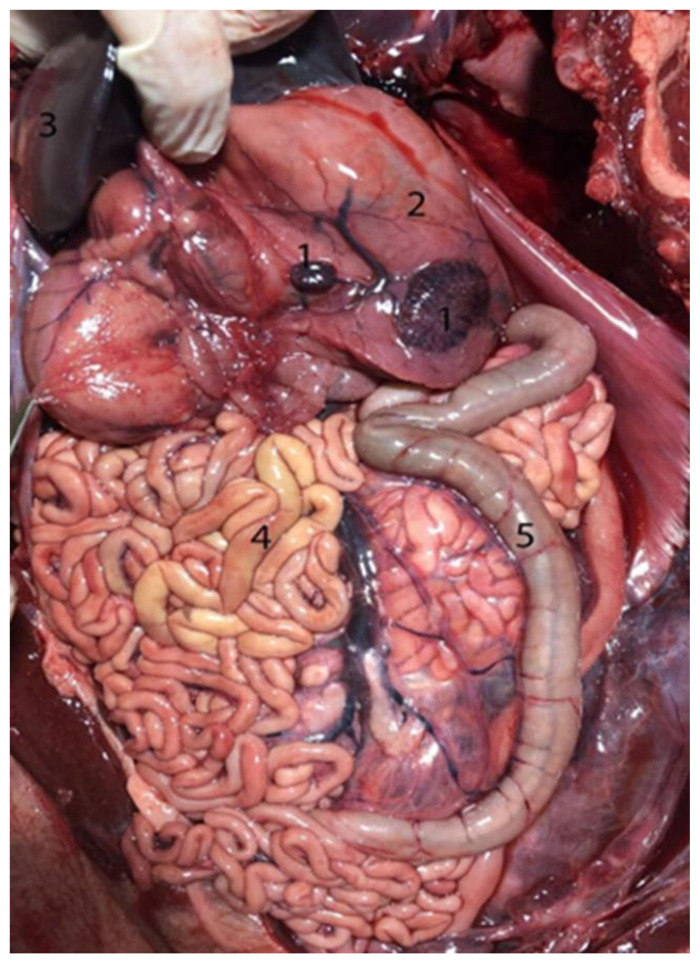
Infant individual. Abdominal cavity. Supine decubitus: (1) spleen and accessory spleen; (2) main stomach; (3) liver; (4) jejunum; (5) ascending colon.

It is associated with the gastrointestinal tract due to its topography, but not functionally. For this reason, it will not be detailed in this study. In several individuals, this organ was not found, possibly due to the degree of decomposition of the animals, its proximity to the pancreas with rapid autodigestion, and its small size (Figure 24). In the two neonate individuals, it could be located on the left side of the abdomen, attached by the gastrosplenic ligament to the main stomach, also in close relation to the pancreas. It is small in size and oval- or button-shaped. Small accessory spleens were also observed (Figure 24).

## 4. Discussion

Among the described organs of the digestive system of the Franciscana dolphin, there are some that share the typical characteristics of the Delfinidae, and these will not be discussed in this paper. However, the focus will be on those that present particular characteristics of this species related to its diet.

First of all, the low skull asymmetry seen in these animals is directly related to its small prey size. The existence of a relationship between prey size and skull asymmetry has been previously described: animals with more asymmetrical skulls are able to capture larger prey than similarly-sized, more symmetrical animals [23,24]. Secondly, because what ultimately determines prey size is the diameter of the maw isthmus and the pharyngeal passage [25,26], the width of the maw isthmus in this study was consistent with the size of the prey consumed: small-sized or juvenile fishes, small marine cephalopods (67% = 11–13 cm mean length), and slightly larger squids [27,28,29,30,31,32].

One might think that the elongated and thin snout of the Pontoporia is a common feature in river species and that it could be related to freshwater life. However, this hypothesis does not hold true for Sotalia, which is also a river dolphin. The authors hypothesize that the Pontoporia, the Amazon dolphin, and the Ganges and Yangtze dolphins probably share a common ancestor, contrary to the Sotalia dolphin, which adapted to life in freshwater much later than the others. In summary, this snout morphology would have more justification from an ontological than an adaptive point of view.

These animals have conical teeth of different lengths, as do other estuarine dolphins, such as the Ganges dolphin (*Platanea platinids*), which are ideal for catching fish and shrimp at an early age [33]. The number of teeth in the upper arch of the Franciscana is similar to that of the lower arch, with 110 pieces on average vs. 108 in juveniles and 113 vs. 110.5 in adults, which differs from those species whose diet is based exclusively on squid and that lack teeth in the upper arch, as explained by Cozzi et al. and Martin Diaz [26,34].

Unlike the tongue of most dolphins [35,36,37], the tongue tip of the Franciscana is barely mobile [38] and very short, even differing from other freshwater dolphins [38]. This is probably compensated by the presence of the anterolateral papillae (anterolateral fimbriae) consisting of compact outgrows placed along a single row developed mainly in infants [35,36,38]. According to Ferrando et al. [39], these latter papillae may help to create a tight seal between the tongue and the roof of the oral cavity and therefore help with suction feeding, as the period of development of these papillae is coincident with the lactation period [40]. Even as we have described in the Franciscana, these papillae tend to be reduced or disappear with age, although they may persist in some individuals [26]. From the age of two months, this species begins to consume solid food and its feeding becomes mixed, and then these papillae assist in holding the prey against the palate while water flows through the papillae [33,34,36,39,41] until they begin to involute due to disuse and completely lose functionality in adulthood.

In the present work, the average number of marginal papillae was 3 in adults, 30 in juveniles, and 50 in neonates. These data are consistent with those of Guimaraes et al. [41], who obtained an average number of marginal papillae of 53 in immature and 36.7 in mature dolphins and only 3.75 on average in adults (being nonexistent in some specimens), but they differ from the 13 to 15 projections counted in juveniles by Yamasaki et al. [38], which was probably measured in older juveniles.

A multiple-chambered stomach is unusual in carnivores. However, all cetaceans are carnivores and have the presence of a multichambered stomach. The Cetartiodactyla shows a tendency towards plurilocular stomachs, which can be found and characterized by very different architectures in Suidae, Tayassuidae, Camelidae, Tragulidae, Pecora, Hippopotamidae, and Cetacea [26,28]. After all, whales are closely related to artiodactyls, which also have multichambered stomachs. While their multiple chambers may relate to the mechanical and enzymatic breakdown of an herbivorous diet (e.g., separation of food to be regurgitated and re-chewed as cud), it is unclear what functions multiple chambers play in the carnivorous cetaceans [24]. Perhaps, in a sense, they compensate for the absence of chewing by grinding and compressing in the first chamber before absorption takes place in the subsequent compartments [26].

While all dolphins share the multicameral anatomy of their stomachs, Mead [42] describes three morphological appearances of the stomach, which he calls the ziphiid stomach (one main stomach, one pyloric stomach), the derived stomach type I (two main stomachs, one pyloric stomach), and the derived stomach type II (two main stomachs, two pyloric stomachs). The Franciscana shows macroscopic features similar to those of a zyphid-type dolphin, with a main stomach, a communicating channel, and a pyloric stomach, as also described by Yamasaki et al. [43].

**Figure 25:**
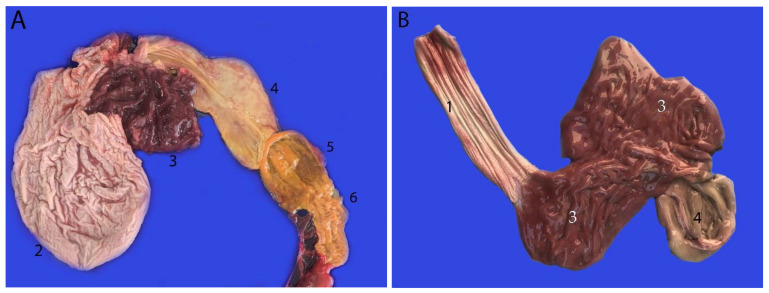
Comparative images of the stomachs of two different dolphins. The esophagus and the fore- and main stomachs are opened to show their mucous: (**A**) multichambered stomach of a *Stenella frontalis* (from IUSA-ULPGC); (**B**) bicameral stomach of a *Pontoporia blainvillei*; (1) esophagus; (2) forestomach; (3) main stomach; (4) pyloric stomach; (5) duodenal ampulla; (6) duodenum.

In the Franciscana, there is no pre-stomach present, as in all known zyphids and contrary to most eutherian mammals (Figure 25). This feature is also thought to be shared with the probably extinct Chinese river dolphin (*Lipotes vexillifer)*, but not so with the Amazon river dolphin (*Inia geoffrensis)*. Marigo and Groch [44] reconfirm that some odontocete species lack an anterior or pre-stomach chamber and, instead, the main chamber is divided into two or three compartments separated by septa [34]. Yet, this is not the case in Franciscana. Storage and mechanical degradation functions have been attributed to the anterior or pre-stomach chamber [25,44,45], which encourages us to think that in the face of the continuous consumption of small prey, this possibility of storage and this reinforcement in digestion for the chemical and physical degradation of the prey are not necessary. With this, the authors say that the storage function is probably being replaced by the increase in feeding frequency (continuous feeder), and, on the other hand, the mechanical action can be performed in the muscular chamber of the main stomach. This structure of the stomach with one less compartment could agree with its antiquity and primitiveness with respect to other cetacean species more evolved or new in the phylogenetic tree. It may also be compensated by the greater length of its small intestine, where enzymatic degradation processes occur.

The stomach values recorded are consistent with the values published by Kamiya and Yamasaki [46]. After the animal has reached a body weight of 20 kg, the relative weight of the stomach at adulthood contributes only approximately 1.5% of the body weight and does not show considerable changes during body growth. While the relative size of the stomach to the live weight of the Franciscana is consistent with values for a strict carnivore of that size [47], the same is not true for the relative size of the intestines.

The small intestine of the Franciscana is similar to that of other carnivores. Yet, the total length of the gut in the Franciscana is remarkably high. The reason for this is the extraordinary length of the jejunum, corroborating what Yamasaki et al. described [20]. According to Crespo (2002), this species feeds mostly near the bottom on fish belonging to several families; it is listed as a predominantly teutophagous odontocete [48]. The reason for the long intestine in Franciscana and the influence of the types of food are not known. Büker, however, gives an interpretation of the lengthening of the intestinal tract in aquatic mammals: during diving, blood supply to the intestines is reduced, and a longer gut could compensate for the necessary assimilation of nutrients [49]. The authors would like to add the possibility of the development of a relatively longer small intestine that allows more time for the food to be in contact with the enzymes that will degrade it, as well as a greater absorption surface in response to a lower-quality diet compared to that of other dolphins, comprising bottom-dwelling juvenile teleosts, squid, and shrimp [31,50,51,52,53,54].

Among the toothed whales, there is considerable variation in the ratio between the length of the small intestine and the body length, ranging from 5/1 in bottlenose whales to 14/1 in some species of dolphin [55]. Baleen whales tend to show lower ratios (fin whales, 4/1; little piked and humpback whales, 5.5/1). These ratios do not necessarily correlate with either the diet or size of the species [56], with the pontoporie ratio being 24–37.7/1.

The small intestine of Franciscana occupies the greater part of the lower abdominal cavity. The length of the small intestine examined by Yamasaki et al. [20] is similar to the values found in this work. However, for this same author, the ratio varied considerably from specimen to specimen, and it seems to show no correlation to body length. Yet, it might have a close relationship with the lapse of time after death rather than individual variation. Also, the length varies in fixed and unfixed states. The length shortened about 8.5% after fixation in 10% formalin solution.

As explained by Cozzi et al. [26] and Marigo and Groch [44] for other species, for the Franciscana, it has been observed that pancreatic juices flow into the duodenum via the pancreatic duct. In most odontocetes, this process is individual, but in some cases, it joins the hepatic duct, thus flowing into what is called an hepatopancreatic duct similar, in this aspect, to equines [34,57], a continuous consumer.

Regarding the arrangement of the jejunal loops in adults, there is a hypothesis that this is in relation to the need to maximize efficiency when taking advantage of the available space. In this way, a longer intestine can be accommodated as time goes by in almost the same abdominal cavity, making space more efficient. And, even as the animals advance in age, their diets become of better quality due to learning how to capture prey and the ecological niches they occupy [51]. This could be consistent with a greater prey metabolism, and therefore less gas production, which allows for a more orderly arrangement of the loops. However, there is no literature to support this hypothesis.

As mentioned in the results, there is not a particular distinction between the small and large intestines, with no caecum or vermiform appendix present. According to the results of this study, the authors agree with Cozzi et al. [26], as this apparently simplified disposition results from the diet, which includes mostly proteins of animal origin (generally fish and cephalopods). The exception to the rule is the Ganges River Dolphin, belonging to the family Platinistidae, which possesses a cecum.

The mucosa of the colon shows one or two longitudinal folds that increase its inner surface and disappear as they move caudally. The Gangetic dolphin, on the other hand, has numerous longitudinal folds that begin in the distal colon and continue to the rectum; in the Amazon river dolphin, the folds appear along the entire colon [28,58,59] Although this fact may be disadvantageous with respect to having circular folds for them to widen the absorption area, the extraordinarily long intestines may compensate for the lack of these type of folds [20,60].

There are differences between our results and previously published data on the total length of the large intestine of the Franciscana dolphin. Apart from the absence of cecum and the similar thickness of both intestines at the junction point, these variances could be related to the criteria used to define the beginning and end of the large intestine, as there are no clear macroscopic signs. In the study by Mead and Brownel, the values obtained were 40–48 cm [61], which were within the range of those described by Yamakasi et al., 25–58 cm [20], and the results of the present study were slightly higher, with 62–81 cm for adults, 45–71 cm for juveniles, and 36–44 cm in infants.

The peculiar morphological characteristics of the gross intestine of dolphins, and specifically the shortage of any bowel-like structure in the gut, could suggest minimal or no storage capacity of undigested residue [62]. In fact, evacuated feces are generally rather liquid and may contain unprocessed materials, especially when shrimps are part of the diet [26]. However, the observation made in some of the studied animals that were under human care was of a more consistent fecal matter than that of other dolphins. This may be related to the greater length of the intestines and therefore the greater surface area of water absorption of the feces, which is consistent with less watery feces.

Regarding the relationship between liver weight and body weight, the average did not include the neonate with hepatomegaly that was found stranded alive and transferred to the rescue center for rehabilitation. In this specimen, the liver weight was 7.5% of the body weight. In association with this high percentage, generalized jaundice was observed in the mucous membranes of this animal. Interestingly, this condition has been observed in many of the neonates that were undergoing rehabilitation at the rescue center Fundación Mundo Marino. This finding is not described in the literature. It is currently being investigated by several Latin American organizations collaborating in the conservation of marine fauna. In the rest of the individuals, the relative values were 2.43%, which coincides with those already described by other authors, where the weight of the liver averages 2–3% of the total body weight in dolphins [21,26,63]. However, these values are higher than the relative values of other cetaceans, with the exception of estuarine dolphins [63], which makes the authors think of a higher metabolic rate in this type of dolphin. These higher metabolic rate data also coincide with the hypothesis expressed by Helm (1983) that the higher the metabolic rate, the longer the relative length of the intestines [64].

The liver of the Franciscana, like the liver of cetaceans and like that of many terrestrial species, lacks a gallbladder, and the bile produced in the hepatic stroma is carried into the duodenum by a common hepatic duct, as mentioned above. The absence of an extra-hepatic storage organ for the bile is possibly related to the continuous ingestion of food and the consequently frequent presence of food in the proximal intestine [26], as is the case in continuous consumers.

## 5. Conclusions

The anatomical characteristics of the digestive system of the Franciscana dolphin are similar to those present in other marine mammals. However, our results show that this dolphin shows several peculiarities, especially in relation to the tongue, the teeth, the stomach, and the small intestine.

Although further studies are needed to confirm the results of the present paper, it could be assessed that the digestive system of the Franciscana dolphin is adapted to a small prey diet, considering the size of its oral cavity, the scarce development of its tongue, and the absence of a pre-stomach that allows for the shredding of larger prey. These animals are continuous consumers. This fact is consistent with the absence of a forestomach and a gall bladder, as well as the presence of a hepatopancreatic duct similar to that of other continuous consumers. The composition of crustaceans and squid in their normal diet could explain the length of their intestine, as it would allow the food to be inside the digestive tract for a longer time to facilitate its enzymatical degradation and absorption.

The importance of this work is clear due to the following reasons. First of all, it is important for anatomists, as there are very few previous descriptions of the anatomy of the digestive system of this dolphin. Then, it is important for physiologists and nutritionists, as anatomy is the basis of a better comprehension of other basic sciences. It is not possible to explain any physiological process without knowing the anatomy of an organ, apparatus, or system. For example, anatomy, digestion, and nutrition are closely related. Once again, the knowledge of the digestive system’s anatomy is critical to understanding certain processes related to the absorption of nutrients, the balance between proteins, fats, and carbohydrates, and metabolism. Therefore, our work supports the idea of anatomy in constant adaptation to nature or external factors to facilitate other processes. And, finally, this paper is also important because the species of interest is in danger, and any knowledge or data about these animals are of relevance and will increase their chances of survival.

In summary, it seems that the protein-based diet of the *Pontoporia blainvillei* could determine the anatomy of its digestive system, although certain phylogenetic adaptations should not be ruled out. Of course, further studies are needed to clarify this hypothesis.

## Figures and Tables

**Table 1:** Morphometric data (number and length) of the teeth of the specimens of *Pontoporia blainvillei* used in the present study.

Age	ID	Maxilla (No)		Jaw (No)		Total (No)	Average (No)	Length (cm)
Group		Right	Left	Right	Left			R-M-C
A	M09	58	59	57	58	232	224	0.5-0.7-0.2
	M43	55	55	53	53	216		0.5-0.7-0.2
J	M20	56	56	51	52	215	218	0.4-0.5-0.2
	M21	52	54	55	53	214		0.4-0.6-0.2
	M60	57	55	53	54	219		0.4-0.5-0.2
	M65	57	57	55	55	224		0.3-0.5-0.2
	M71	52	52	53	52	209		0.5-0.6-0.4
	M78	59	58	56	56	229		0.4-0.5-0.2

A: adult; J: juvenile; ID: individual identification; R-M-C: rostral zone—middle zone—caudal zone of maxilla/mandible.

**Table 2:** Morphometric data of the tongue and the esophagus of the specimens of *Pontoporia blainvillei* used in the present study.

		Tongue					Esophagus				
Age	ID	Weight	Length	Width (cm)		Papillae m.	Weight	Length	Diameter (cm)		
Group		(g)	(cm)	Base	Vertex	(No)	(g)	(cm)	cr	med	cd
A	M09	36	6	3.2	1.5	9	51	27	3.5	2.5	3
	M22	47	7	4	2	0	163	29	5	3.5	4.5
	M24	39	6.5	3.7	1.8	0	192	35	6.2	4.4	4.7
	M43	34	6	3.5	1.9	0	160	33	6	4	4.5
	M47	31	5	3.5	2	6	135	32	5	3	3.3
	mean	37	6.1	3.6	1.8	3.0	140	31	5.1	3.5	4.0
J	M12	28	4.3	2.5	1.9	37	19	19	2.4	---	1.5
	M16	35	5.7	3.6	2.1	27	77	26	3.5	---	2.8
	M20	31	5.7	3.5	1.7	29	92	26	4	3.2	3.6
	M21	27	5.5	3	1.4	15	95	24	2.8	2.4	2.6
	M23	28	5.5	2.8	2	38	44	20	3	1.6	2.5
	M28	33	6	3	2	19	63	24	3	2.5	2.8
	M29	27	6	2.8	2	25	38	23	2.8	2.2	2.5
	M45	27	6	2.9	2.4	32	67	29	3	2.4	2.6
	M60	25	6	2.8	2	41	40	24	2.2	---	1.8
	M71	28	6	2.5	2	35	59	27	2.8	---	3.2
	M78	34	6.7	3.2	2.4	30	116	31	4.3	3.7	4
	mean	29	5.8	3	2.0	30	65	25	3.1	2.6	2.7
N	M17	25	5.5	2.5	2	51	21	18	2	1.4	1.8
	M81	22	5	2.3	1.7	48	12	15	1.5	0.9	1.3
	mean	24	5.3	2.4	1.9	50	17	17	1.8	1.1	1.6

A: adult; J: juvenile; N: neonate; ID: individual identification; papillae m.: mechanical papillae; cr: cranial; med: medium; cd: caudal.

**Table 3:** Morphometric data of the main and pyloric stomachs of the specimens of *Pontoporia blainvillei* used in the present study.

Age	ID	Weight (g)	Capacity (mL)		Curvature MS (cm)		Length (cm)		Body Width (cm)		Fundus Width
Group		MS	MS	PS	Major	Minor	MS	PS	MS	PS	MS
A	M09	290	1150	105	39	13	15	9	9	5	7
	M22	489	1450	100	44	15	18	9.5	12	4	8
	M24	714	2100	245	46	17	21	10	14	5.5	9
	M43	575	1870	195	43	15	17	9.5	10	5	9
	M47	410	1600	170	40	14	16	9.4	10	4	7
	mean	495	1634	163	42	15	17	9	11	4.7	8
J	M12	96	140	16	29	9	10	5	8	3	6
	M16	281	800	90	36	16	19	10	14	4	8
	M20	380	1330	155	37	17	20	9	12	4	9
	M21	324	1200	120	38	13	17	11	11	4.5	7
	M23	136	320	40	27	6	10	6	7	3	5
	M28	327	820	60	38	15	14	8	9	4	7
	M29	136	400	65	30	9	11	5	9	3	6
	M45	265	790	105	31	11	18	6	10	4	8
	M60	110	290	25	28	8	11	6	7	4	6
	M71	230	600	75	26	11	15	5	10	4	7
	M78	385	940	105	32	13	22	6	15	4	10
	mean	243	694	78	32	12	15	7	10	4	7
N	M17	60	130	20	19	7	9	4	6	3.5	4
	M81	25	60	12	7	4	5	1.8	4	1.8	3
	mean	43	95	16	13	5.5	7	2.9	5	2.7	3.5

A: adult; J: juvenile; N: neonate; ID: individual identification; MS: main stomach; PS: pyloric stomach.

**Table 4:** Morphometric data of the small intestine of the specimens of *Pontoporia blainvillei* used in the present study.

Age	ID	Weight (g)			Length (cm)				Diameter (cm)					
					D	AD	J	I	Duodenum		AD	Jejunum		Ileum
Group		D	J	I	(cm)	(cm)	(m)	(cm)	cr	cd		cr	cd	
A	M09	15	1110	---	9	5	35	---	1.7	1.3	4	1.1	0.9	---
	M22	21	1200	7	10	5	37	9	2	1.5	4	1.2	1	1
	M24	24	1720	9	11	5	38	10	3	1.7	5	1.5	1.4	1
	M43	15	1040	6	14	4.5	37	11	2.6	1.4	4	1.5	1.3	1.1
	M47	10	930	5	14	5	36	8	3.2	1.5	4	1.4	1.2	1
	mean	17	1200	6.8	11.6	4.9	37	9.5	2.5	1.5	4.2	1.3	1.2	1.0
J	M12	6.5	200	---	8	3	16	---	2	1	3	0.5	0.4	---
	M16	14	850	---	12	4	34	---	1.6	1.1	3	0.8	0.7	---
	M20	11	1130	5	9	3	32	8	2.5	1.5	4	1.1	0.8	1
	M21	13	800	2.7	11	4	31	7	2	1	4	0.8	0.6	1
	M23	6.4	530	1.4	7	3	23	5	1.4	1	3.5	0.9	0.7	0.6
	M28	12.6	1000	2.5	11	5	32	4	2	1.2	4	1	0.8	0.9
	M29	6,2	485	1.2	9	4	22	4	2	1	4	0.9	0.7	0.6
	M45	18	750	3.5	10	5	31	5	2.2	1.2	4	0.8	0.7	0.7
	M60	5	310	2	8	---	21	6	---	---	---	0,7	0.6	1
	M71	22	400	---	10	5	25	---	2.3	1.4	5	0.8	0.7	---
	M78	20	640	---	9	7.5	30	---	2	1.2	5.5	0.9	0.7	---
	mean	12.2	672	2.7	9.5	4.4	27	5.9	2.0	1.2	4.0	0.8	0.7	0.9
N	M17	3	180	1.8	7	2.5	15	4	0.8	0.5	2	0.4	0.4	0.5
	M81	1	105	1	6	1.4	13	4	0.6	0.4	1.2	0.4	0.4	0.4
	mean	2	142	1.4	6.5	2.0	14	4	0.7	0.5	1.6	0.4	0.4	0.45

A: adult; J: juvenile; N: neonate; ID: individual identification; D: duodenum; AD: duodenal ampulla; J: jejunum; I: ileum; cr: cranial; cd: caudal.

**Table 5:** Morphometric data of the large intestine of the specimens of *Pontoporia blainvillei* used in the present study.

Age	ID	Colon				Straight
		Weight	Length	Diameter (cm)		Length
Group		(g)	cm	cr	cd	cm
A	M09	37	62	1.7	1.5	3
	M22	49	64	1.7	1.4	4
	M24	60	81	2.2	1.6	5
	M43	41	67	2	1.3	4
	M47	54	76	1.8	1.2	4
	mean	17	70	1.9	1.4	4
J	M12	28	45	1	0.7	3
	M16	49	51	1.3	0.8	3.5
	M20	37	55	1.1	1.6	4
	M21	60	68	1.2	1.4	2
	M23	42	48	1.6	1.3	3
	M28	50	65	1.8	1.3	4
	M29	43	60	1.6	1.3	4.5
	M45	42	63	1.6	1.4	4
	M60	30	51	1.4	1.1	3
	M65	36	52	1.4	1.2	3
	M71	30	61	1.6	1.4	4
	M78	45	71	1.7	1.4	5
	mean	41	58	1.4	1.2	3.6
N	M17	17	44	0.8	0.6	2.5
	M81	8	36	0.6	0.5	1.5
	mean	13	40	0.7	0.6	2

A: adult; J: juvenile; N: neonate; ID: individual identification; cr: cranial; cd: caudal.

**Table 6:** Measures of the liver weight of the specimens of *Pontoporia blainvillei* used in the present study.

Age	ID	Liver Weight (g)			Body Weight (kg)	Ratio Liver/Body Weight (%)
Group		Total	Right Lobe	Left Lobe		
A	M09	455	277	178	20.6	2.2
	M22	510	326	184	42.3	1.2
	M24	1240	730	510	61.4	2
	M43	700	415	285	38.5	1.8
	M47	665	400	65	27	2.4
	mean	714	430	244	38	1,9
J	M12	220	130	90	9.2	2.4
	M16	196	140	56	18.3	1
	M20	526	332	194	22.2	2.4
	M21	506	357	149	23.8	2.1
	M23	277	174	103	10.6	2.6
	M28	440	287	153	16.2	2.7
	M29	327	228	99	12.5	2.6
	M45	380	240	140	14.7	2.5
	M60	245	140	105	10	2.4
	M65	250	150	100	14.5	1.7
	M71	240	140	100	13.2	2.4
	M78	545	365	180	19.2	2.8
	mean	366	235	131	15	2.4
N	M17	310	135	175	4.1	7.5
	M81	115	70	45	3.9	2.9
	mean	115	70	45	4.0	2.9

A: adult; J: juvenile; N: neonate; ID: individual identification; cr: cranial; cd: caudal. M17: individual with hepatomegaly, not included in the mean liver weight.

## Data Availability

All data concerning this research are include in the Tables of the present manuscript.
